# Difficult patients in mental health care–who are they?

**DOI:** 10.1192/j.eurpsy.2023.1907

**Published:** 2023-07-19

**Authors:** I. M. Figueiredo, G. Soares, C. Lopes, A. C. Rodrigues, A. L. Falcão, A. Lourenço, I. Cargaleiro, M. Nascimento, C. Oliveira

**Affiliations:** 1 Hospital Centre of Psychiatry of Lisbon, Lisbon, Portugal

## Abstract

**Introduction:**

Difficult patients are not something new and we can find innumerous definitions for this concept. However, they form a very heterogenous group and we need a less abstract definition focused more on the clinical reality and the difficulties experienced by patients and mental health professionals.

**Objectives:**

Our goal was to find a more precise and clinical definition of the difficult patient based on quantitative measures using a statistical analysis of a series of hospitalizations.

**Methods:**

A cluster analysis of our hospital’s in-patient treatment from the last 5 years was made concerning the duration of the stay and the number of previous hospitalizations.

**Results:**

A sample of 8576 inpatient treatment episodes was used. 52.4% were male and 47.6% female patients between the age of 15 and 103 years old. The length of the treatment varied from 0 to 1007 days and the number of previous hospitalizations from 0 to 109; excluding the outliers the means were, respectively, 21 days and 2 previous hospitalizations.

The cluster analysis excluded 85 episodes and it found the presence of 3 clusters, being the number 1 the wider one (n=5861 episodes) and the other quite similar.

The Cluster 1 was characterized by a smaller length of hospital stay and number of hospitalizations; the Cluster 2 was defined by the episodes with the highest number of previous hospitalizations (`x =8.77) and the Cluster C by the longest hospital stays (`x =58.09 days). With a Kruskal-Wallis test we found both variables statistically different between all clusters (p<0.001). In Cluster 2 and 3, respectively, we found that 40,24% and 34.61% was taking the medication before being hospitalized, 6.42% and 3.15% were compulsive hospitalizations, and 40.5% and 21.89% had LAI prescribed.

Concerning the diagnosis, Cluster 1 had more Depression, Neurotic and Somatoform disorders; Cluster 2 more Bipolar and Intellectual disability disorders and Cluster 3 more Dementia and Delusional disorders. Substance use disorders and Personality disorders were found more common in both Cluster 1 and 2, Schizophrenia in Cluster 2 and 3 and Psychosis non specified in Cluster 1 and 3.

**Conclusions:**

We can say Cluster 1 comprises the non-difficult patients and it’s not surprising that it includes more Depression and Neurotic and Somatoform disorders. The other diagnostic distributions among clusters were also expected and we can also theorize that Cluster 3 had higher percentages of social cases. Treatment with LAI is linked to a decrease in rehospitalizations and we found that in the majority of these episodes it wasn’t been applied. This research is important in order to identify the difficult patients and what challenges they can bring to the mental health services. Creating these patients’ profile will allow us to better understand their needs to create guidelines for a personalized inpatient treatment and to improve community services to prevent the rehospitalizations and prolonged hospital stays.

**Disclosure of Interest:**

None Declared

